# HDAC3 Regulates Ferroptosis via Nrf2–GPX4 Signaling in Colorectal Cancer Cells

**DOI:** 10.1134/S1607672925600496

**Published:** 2025-09-14

**Authors:** Wei Jin, Jue-jue Wang, Yan-fei Feng, Bing Chen, Zhao-hua Hu

**Affiliations:** 1 Department of Nursing, The Second Affiliated Hospital of Zhejiang University School of Medicine, Zhejiang, Hangzhou, 310000 Zhejiang, China; 2 Department of Vascular Surgery, The Second Affiliated Hospital, Zhejiang University School of Medicine, Zhejiang Province, 310009 Hangzhou, China

**Keywords:** colorectal cancer, HDAC3, ferroptosis, NRF2, GPX4

## Abstract

Ferroptosis, an iron-dependent form of regulated cell death, represents an emerging therapeutic vulnerability in colorectal cancer (CRC). However, the epigenetic mechanisms controlling ferroptosis sensitivity in CRC remain poorly understood. Here, we identify histone deacetylase 3 (HDAC3) as a pivotal epigenetic suppressor of ferroptosis. Both pharmacological inhibition and genetic knockdown of HDAC3 significantly enhanced ferroptosis sensitivity, as evidenced by elevated intracellular ferrous iron (Fe^2+^) and lipid peroxidation. Mechanistically, inhibition of HDAC3 reduced the expression of nuclear factor erythroid 2–related factor 2 (NRF2), a master antioxidant transcription factor, thereby leading to downregulation of glutathione peroxidase 4 (GPX4), a central ferroptosis defense gene. Notably, NRF2 knockdown abolished GPX4 downregulation by HDAC3 inhibition, whereas GPX4 overexpression rescued the ferroptotic phenotype caused by HDAC3 depletion. Collectively, these findings define an HDAC3–NRF2–GPX4 axis that suppresses ferroptosis in CRC, and highlight HDAC3 as a potential therapeutic target for ferroptosis-based cancer treatment.

**Core tip:** This study reveals HDAC3 as a key epigenetic regulator that inhibits ferroptosis in colorectal cancer through modulation of the NRF2–GPX4 signaling axis. We show that suppressing HDAC3—either pharmacologically or genetically—leads to decreased NRF2 transcription, reduced GPX4 expression, and elevated ferroptotic cell death. Increased intracellular iron accumulation upon HDAC3 inhibition further underscores its role in ferroptosis resistance. Functional rescue assays demonstrate that GPX4 is essential for mediating the ferroptosis-sensitizing effects of HDAC3 depletion. Collectively, these findings highlight the HDAC3–NRF2–GPX4 pathway as a promising therapeutic target to enhance ferroptosis susceptibility in colorectal cancer.

## INTRODUCTION

Colorectal cancer (CRC) is a leading cause of cancer-related^1^ morbidity and mortality worldwide [1]. Despite advances in targeted therapies and immunotherapy, resistance to regulated forms of cell death—such as apoptosis and ferroptosis—continues to undermine treatment efficacy [2]. Ferroptosis is an iron-dependent form of regulated necrosis, characterized by the accumulation of lipid peroxides, and has emerged as a promising therapeutic vulnerability across various malignancies, including CRC [3]. Inducing ferroptosis in tumor cells offers a potential strategy to eliminate therapy-resistant populations [4].

The sensitivity of cancer cells to ferroptosis is tightly regulated by metabolic and transcriptional networks [5]. Among them, glutathione peroxidase 4 (GPX4) plays a central role by reducing lipid hydroperoxides and maintaining redox homeostasis [6]. Aberrant overexpression of GPX4 in cancer cells enhances resistance to ferroptosis and supports tumor survival under oxidative stress [7]. Thus, elucidating the upstream regulatory mechanisms that control GPX4 expression is essential for manipulating ferroptosis sensitivity in CRC.

Recent studies have implicated epigenetic regulators—including histone deacetylases (HDACs)—in the modulation of ferroptosis through transcriptional control of ferroptosis-related genes [8]. HDAC3, a class I HDAC, functions as a transcriptional corepressor and has been linked to poor prognosis and chemoresistance in CRC and other tumors [9]. However, its role in ferroptosis regulation remains largely unexplored.

In this study, we performed a pharmacologic screen of HDAC inhibitors and identified HDAC3 as an epigenetic regulator that suppresses ferroptosis in CRC cells. Inhibition or knockdown of HDAC3 sensitized CRC cells to ferroptotic stimuli and increased intracellular iron and lipid ROS levels. Mechanistically, inhibition of HDAC3 reduced the expression of NRF2, a master transcription factor governing antioxidant responses, thereby leading to downregulation of GPX4. Silencing NRF2 abrogated GPX4 downregulation caused by HDAC3 inhibition, while overexpression of GPX4 rescued ferroptosis sensitivity in HDAC3-deficient cells.

Together, these findings uncover an epigenetic regulatory axis—HDAC3–NRF2–GPX4—that governs ferroptosis in CRC and propose HDAC3 as a potential therapeutic target for sensitizing tumors to ferroptosis-inducing treatments.

## MATERIALS AND METHODS

### Cell Culture

HCT116 human colorectal cancer cells were obtained from ATCC and cultured in Dulbecco’s Modified Eagle Medium (DMEM; Gibco) supplemented with 10% fetal bovine serum (FBS; Gibco) and 1% penicillin-streptomycin (HyClone). Cells were maintained in a humidified incubator at 37°C with 5% CO_2_. The culture medium was changed every 2–3 days, and cells were passaged using 0.25% trypsin-EDTA (Gibco) when they reached approximately 80% confluency. Cells were routinely tested and confirmed to be mycoplasma-free.

For specific experiments, cells were treated with HDAC inhibitors including TSA (pan-HDAC), SAHA (pan-HDAC), MS-275 (Class I), RGFP966 (HDAC3-specific), and Tubastatin A (HDAC6-specific), as well as the ferroptosis inducer RSL3 and the ferroptosis inhibitor ferrostatin-1 (Fer-1), as described in the corresponding figure legends.

### Drug Treatments

For pharmacological experiments, cells were treated with the following compounds for 24 h unless otherwise specified:

– Trichostatin A (TSA; APExBIO, Cat# A8183, USA) was used as a pan-HDAC inhibitor at a final concentration of 500  nM.

– Suberoylanilide hydroxamic acid (SAHA; Beyotime, Cat# SC0231-10 mM) was used as a pan-HDAC inhibitor at a final concentration of 1  μM.

– MS-275 (Entinostat; Beyotime, Cat# SD1083-25mg) was used as a Class I HDAC inhibitor at a final concentration of 2  μM.

– RGFP966 (Selleck Chemicals, Cat# S7229) was used as an HDAC3-selective inhibitor at a final concentration of 10  μM.

– Tubastatin A (Beyotime, Cat# SD1155-25 mg) was used as an HDAC6-selective inhibitor at a final concentration of 10  μM.

– RSL3 (Yeasen Biotech, Cat# 53469ES08) was used as a ferroptosis inducer at a final concentration of 1  μM.

– Ferrostatin-1 (Fer-1; APExBIO, Cat# A4371) was used as a ferroptosis inhibitor at a final concentration of 1  μM.

All compounds were dissolved in DMSO. The final DMSO concentration did not exceed 0.1% in any treatment condition.

### siRNA and shRNA Transfection

**siRNA–mediated knockdown of transcription factors.** Predesigned siRNAs targeting human NRF2, SP1, ELK1, and CREB were purchased from GenePharma (Suzhou, China). Cells were transfected with Lipofectamine RNAiMAX (Thermo Fisher Scientific) at a final siRNA concentration of 50 nM, following the manufacturer’s instructions. Knockdown efficiency was evaluated by qPCR at 72 h post-transfection, achieving >70% reduction in mRNA levels.

**shRNA–mediated stable knockdown of HDAC3.** A validated shRNA targeting human HDAC3 (sequence: 5′‑CCTTCCACAAATACGGAAATT‑3′) was obtained from the Sigma MISSION TRC library (Clone TRCN0000004825). The shRNA oligo was cloned into the pLKO.1 vector. Lentiviral particles were produced in HEK293T cells via co-transfection with psPAX2 and pMD2.G packaging plasmids using PEI. Viral supernatants were collected at 48 and 72 h, filtered, and used to infect HCT116 cells in the presence of 8 µg/mL polybrene. After 24  h, cells were selected with 2 µg/mL puromycin for 7 days to establish stable knockdown lines. Knockdown efficiency (>80%) was confirmed by both qPCR and Western blot.

**GPX4 overexpression.** A commercially sourced human GPX4 cDNA plasmid encoding a C‑terminal FLAG^®^ tag in the pcDNA3.1 backbone (Sino Biological, Cat. No. HG18231‑CF) was used. HCT116 cells were seeded in 6‑well plates and transfected with 1 µg of the purchased plasmid per well using Lipofectamine^®^ 2000, following the manufacturer’s protocol. Cells were harvested 48 h post‑transfection for Western blotting and downstream functional assays.

**Cell viability assay. CCK-8 assay:** Cell viability was assessed using the Cell Counting Kit-8 (CCK-8; Dojindo Molecular Technologies) according to the manufacturer’s protocol. HCT116 cells were seeded in 96-well plates at a density of 5 × 10^3^ cells/well and allowed to adhere overnight.

Cells were then treated with RGFP966 (10 µM, HDAC3-specific inhibitor), RSL3 (1 µM, ferroptosis inducer), or their combination for 24 or 48 h, as specified in figure legends. For rescue experiments, cells were transfected with either a GPX4-overexpression plasmid or empty vector 24 h prior to compound treatment. In some assays, Ferrostatin-1 (Fer-1, 1 µM) was co-administered to block ferroptosis.

Following treatment, 10 μL of CCK-8 reagent was added to each well and incubated at 37°C for 2 h. Absorbance was measured at 450 nm using a microplate reader (Bio-Rad). All conditions were tested in triplicate, and results are expressed as mean ± standard deviation (SD) from at least three independent experiments.

### Quantitative Real-Time PCR (qPCR)

**RNA isolation and cDNA synthesis.** Total RNA was extracted from HCT116 cells using TRIzol reagent (Invitrogen, USA) according to the manufacturer’s instructions. RNA concentration and purity were assessed using a NanoDrop spectrophotometer. For cDNA synthesis, 1 μg of total RNA was reverse-transcribed using the PrimeScript RT Reagent Kit (TIANGEN, China).

**qPCR analysis.** Real-time PCR was performed using a SYBR Green Master Mix (Takara, Japan) on a real-time PCR detection system (e.g., from Bio‑Rad). Each 20  μL reaction contained 10  μL SYBR Green mix, 1 μL of forward primer, 1 μL of reverse primer, 1 μL of cDNA template, and 7  μL of nuclease‑free water. The cycling program was: 95°C for 5 min, followed by 40 cycles of 95°C for 10 s, 60°C for 20 s, and 72°C for 30 s.

Gene expression was normalized to GAPDH, and relative mRNA levels were calculated using the 2^-ΔΔCt method. The following primer sequences were used:

COX2 (PTGS2):

Forward: AGACACTCTATCACTGGCAC

Reverse: TCTGTACTGCGGGTGGAACA

ACSL4:

Forward: TCCAGACATCCAGAGACCAA

Reverse: CTTCTCGGCTTCCTCCATGT

PTGS2:

Forward: TGAGCATCTACGGTTTGCTG

Reverse: GCTCGGCTTCCAGTATTGAG

NOX1:

Forward: GACAGAGGGCTTTGCATCTG

Reverse: CGTTCACCTTCCTCTGGATG

FTH1:

Forward: AGCCACATTCATCGGCTACA

Reverse: GATGTGGAGCTTGGACATGC

SLC7A11:

Forward: GGAGGAGCCCAAGATCGTGAA

Reverse: CTCCACACTGAAGGCATCGT

GPX4:

Forward: TGTGAGGCAAGACCGAAGT

Reverse: TGGTCTTGGCGTTCTCCTG

GAPDH (internal control):

Forward: GGAGCGAGATCCCTCCAAAAT

Reverse: GGCTGTTGTCATACTTCTCATGG

### Western Blot Analysis

**Protein extraction and quantification.** HCT116 cells were lysed in RIPA buffer (Beyotime, China) supplemented with protease and phosphatase inhibitor cocktails (Beyotime). Lysates were incubated on ice for 30  min, then centrifuged at 12 000 × g for 15 min at 4°C. Supernatants were collected as total protein extracts. Protein concentrations were determined using a BCA Protein Assay Kit (Beyotime), according to the manufacturer’s instructions.

**Western blotting.** Equal amounts of protein (25–30  μg per sample) were separated by 10% SDS-PAGE, then transferred onto PVDF membranes (Millipore, IPVH00010). Membranes were blocked in 5% non-fat milk dissolved in TBS-T (20  mM Tris-HCl, 150  mM NaCl, 0.1% Tween-20, pH 7.6) for 1 h at room temperature, and subsequently incubated overnight at 4°C with primary antibodies diluted in blocking buffer (1 : 1000 unless otherwise specified):

HDAC3 (Rabbit mAb, D2O1K, Cell Signaling Technology, #85057)

GPX4 (Rabbit pAb, Abcam, #ab125 066)

NRF2 (Rabbit pAb, Proteintech, #16 396-1-AP)

β-Actin (Mouse mAb, Proteintech, #66 009-1-Ig)

The next day, membranes were washed and incubated with HRP‑conjugated secondary antibodies (Beyotime, China) for 1 hour at room temperature. Signal detection was carried out using ECL substrate (Thermo Fisher, #32 106), and chemiluminescence images were captured using a gel documentation system. Band intensities were quantified with ImageJ software and normalized to β-Actin as a loading control.

**Intracellular iron measurement.** Intracellular ferrous iron (Fe^2+^) levels were quantified using the Iron Assay Kit (Sigma‑Aldrich, Cat# MAK025) according to the manufacturer’s protocol. In brief, HCT116 cells were seeded in 6‑well plates at 3  ×  10^5^ cells/well and treated with HDAC inhibitors and/or ferroptosis modulators (e.g., RSL3, Fer‑1) for 24 h. After treatment, cells were washed twice with cold PBS and lysed in 200 μL of the kit’s iron assay buffer. Lysates were cleared by centrifugation at 13 000 *g* for 10 min at 4°C. For Fe^2+^ measurement, 50  μL of supernatant was transferred into a 96‑well plate and mixed with the iron detection reagent (without reducing agent). After a 60‑min incubation at room temperature in the dark, absorbance was read at 593 nm on a microplate reader. Fe^2+^ levels were normalized to total protein concentration, determined using the BCA Protein Assay Kit (Beyotime) from parallel lysates. All conditions were assayed in technical triplicates, and each experiment was independently repeated at least three times.

**Reactive oxygen species (ROS) detection.** Intracellular reactive oxygen species (ROS) levels were measured using CellROX™ Green Reagent (Invitrogen, Cat# C10444), in accordance with the manufacturer’s protocol. HCT116 cells were seeded onto sterilized glass coverslips placed in 24-well plates (approximately 5 × 10^4^ cells/well) and allowed to adhere overnight. Cells were then treated with HDAC inhibitors or vehicle controls for 24 h.

Following treatment, cells were incubated with 5 μM CellROX Green Reagent diluted in pre-warmed complete medium at 37°C for 30 min in the dark. After staining, cells were gently washed twice with PBS, then fixed in 4% paraformaldehyde (PFA) for 15 min at room temperature.

Coverslips were mounted using DAPI-containing antifade mounting medium (e.g., ProLong™ Gold, Invitrogen) and imaged using a fluorescence microscope (excitation/emission: 485/520 nm for CellROX; DAPI channel for nuclei). ROS fluorescence intensity was quantified using ImageJ software in at least five randomly selected microscopic fields per sample. Data were averaged across three independent biological replicates.

**Statistical analyses.** All quantitative results are presented as mean ± standard deviation (SD), based on at least three independent biological replicates unless otherwise stated. Statistical comparisons between two groups were performed using unpaired two-tailed Student’s t-test, while comparisons among three or more groups were analyzed by one-way analysis of variance (ANOVA) followed by Tukey’s multiple comparisons post hoc test.

A *p*-value < 0.05 was considered statistically significant. All statistical analyses were conducted using GraphPad Prism 9.0 (GraphPad Software, San Diego, CA, USA).

For Western blot quantification, densitometric analysis was carried out using ImageJ software (NIH), and protein expression levels were normalized to GAPDH or β-actin as internal controls. The number of replicates (*n*), type of statistical test used, and exact *p*-values (where applicable) are reported in the corresponding figure legends.

## RESULTS

### Class I HDAC Inhibition Sensitizes Colorectal Cancer Cells to Ferroptosis

To investigate the role of histone deacetylases (HDACs) in modulating ferroptosis in colorectal cancer (CRC), HCT116 cells were treated with a panel of HDAC inhibitors targeting different HDAC classes. These included the pan-HDAC inhibitors trichostatin A (TSA) and SAHA, the Class I-selective inhibitor MS-275, the HDAC3-specific inhibitor RGFP966, and the HDAC6-selective inhibitor Tubastatin A. After 24 h of treatment, intracellular ferrous iron (Fe^2+^) and reactive oxygen species (ROS) levels were measured to evaluate ferroptotic activity.

**Fig. 1.**
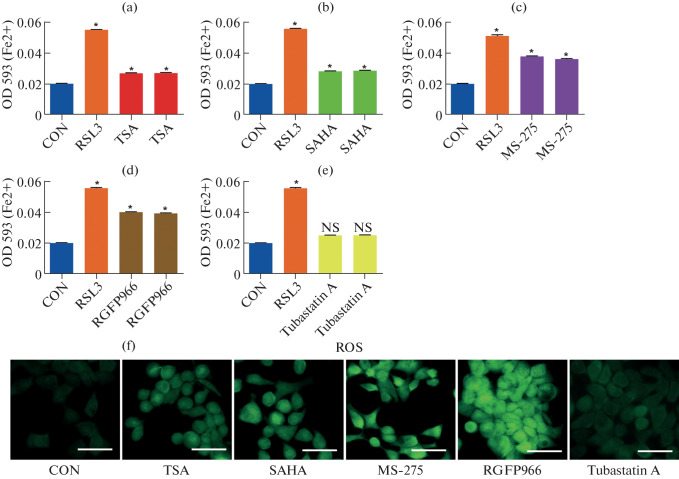
Class I HDAC inhibition enhances ferroptosis sensitivity in colorectal cancer cells. (a–e) HCT116 cells were treated with the ferroptosis inducer RSL3 (1  µM) in combination with various HDAC inhibitors for 24  h. Intracellular ferrous iron (Fe^2+^) levels were quantified using the Iron Assay Kit (MAK025, Sigma-Aldrich). Each treatment group consisted of three technical replicates from a representative experiment; data are mean ± SD. Statistical significance was determined by one‑way ANOVA with Tukey’s post hoc test (*p < 0.05 vs CON; NS = not significant). Bars are color‑coded: **blue** = control (CON), **orange** = RSL3, **red** = TSA, **green** = SAHA, **purple** = MS‑275, **brown** = RGFP966, **yellow** = Tubastatin A. (a) TSA (500  nM, pan‑HDAC inhibitor) significantly increased Fe^2+^ accumulation. (b) SAHA (1  µM, pan‑HDAC inhibitor) significantly increased Fe^2+^ accumulation. (c) MS‑275 (2  µM, Class I HDAC inhibitor) markedly increased Fe^2+^ levels. (d) RGFP966 (10  µM, HDAC3‑specific inhibitor) strongly enhanced Fe^2+^ levels, suggesting HDAC3 involvement. (e) Tubastatin A (10 µM, HDAC6‑selective inhibitor) showed no significant effect. (f) Reactive oxygen species (ROS) levels were visualized using CellROX™ Green; representative fluorescence images are shown. Scale bar = 50 µm.

As shown in Figs. 1a–1e, treatment with TSA (red), SAHA (green), MS-275 (purple), and RGFP966 (brown) significantly increased Fe^2+^ accumulation compared to control cells (blue). Among these, MS-275 and RGFP966 produced the strongest effects, suggesting a specific role for Class I HDACs in regulating iron homeostasis during ferroptosis. In contrast, Tubastatin A (yellow), a Class IIb HDAC6 inhibitor, showed no significant effect, indicating a class-specific involvement of HDACs in ferroptotic regulation.

To assess oxidative stress, fluorescence imaging of intracellular ROS was performed using a selective fluorescent probe. Consistent with the iron measurements, TSA, SAHA, MS-275, and RGFP966 markedly increased ROS accumulation, while Tubastatin A had minimal impact (Fig. 1f). A scale bar was included in the images to indicate magnification.

Collectively, these results suggest that Class I HDACs act as negative regulators of ferroptosis in CRC cells. Among them, HDAC3 inhibition by RGFP966 produced the most pronounced ferroptotic phenotype, highlighting HDAC3 as a potential epigenetic target for sensitizing CRC cells to ferroptosis-inducing therapies.

### HDAC3 Positively Regulates GPX4 Expression to Suppress Ferroptosis in Colorectal Cancer Cells

**Fig. 2.**
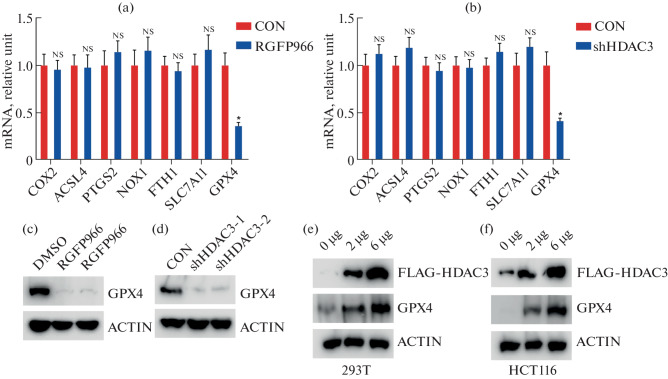
HDAC3 regulates GPX4 expression in colorectal cancer cells. (a, b) Quantitative PCR analysis of ferroptosis-related gene expression in HCT116 cells treated with RGFP966 (10 µM, 24 h) (a) or subjected to lentiviral shRNA-mediated HDAC3 knockdown (b). Among the tested genes (COX2, ACSL4, PTGS2, NOX1, FTH1, SLC7A11, and GPX4), only GPX4 mRNA was significantly reduced upon HDAC3 inhibition or silencing (*p < 0.05), while others remained unchanged (NS, not significant). Data represent mean ± SD from three independent experiments. (c, d) Western blot analysis showing decreased GPX4 protein levels following RGFP966 treatment (c) or HDAC3 knockdown using two independent shRNAs (d). ACTIN served as a loading control. Blots are representative of three independent experiments. (e, f) Overexpression of HDAC3 using FLAG-tagged plasmid in 293T (e) and HCT116 (f) cells induced a dose-dependent increase in GPX4 protein expression.

To elucidate the mechanistic link between HDAC3 and ferroptosis, we first evaluated the expression of key ferroptosis-related genes following HDAC3 inhibition or knockdown in HCT116 cells. Quantitative PCR analysis revealed that among the genes assessed (COX2, ACSL4, PTGS2, NOX1, FTH1, SLC7A11, and GPX4), only GPX4 mRNA levels were significantly reduced upon HDAC3 inhibition with RGFP966 or HDAC3 silencing using two independent shRNAs (Figs. 2a–2b).

Consistent with these transcriptional changes, Western blot analysis confirmed a marked reduction in GPX4 protein levels following either RGFP966 treatment or HDAC3 knockdown (Figs. 2c–2d). Conversely, overexpression of HDAC3 in both 293T and HCT116 cells led to a dose-dependent increase in GPX4 protein abundance (Figs. 2e–2f), supporting a positive regulatory role of HDAC3 in maintaining GPX4 expression.

These results demonstrate that HDAC3 sustains GPX4 expression at both the mRNA and protein levels, suggesting that HDAC3 may act as a repressor of ferroptosis in colorectal cancer cells by regulating GPX4 transcriptionally and post-transcriptionally.

### NRF2 Is Required for HDAC3-Mediated Regulation of GPX4 Expression

To investigate the mechanism by which HDAC3 regulates GPX4 expression, we hypothesized that HDAC3 may act through transcription factors known to control GPX4 transcription. Based on promoter analysis and previous studies, we selected NRF2, SP1, ELK1, and CREB as candidate transcription factors for functional screening.

**Fig. 3.**
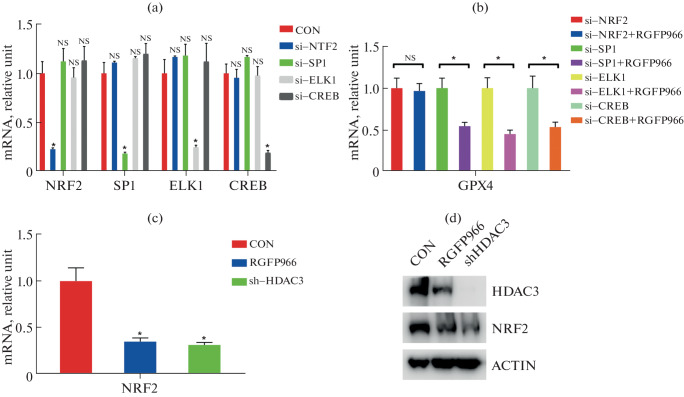
HDAC3 regulates GPX4 expression via NRF2 in colorectal cancer cells. (a) qPCR analysis confirming siRNA-mediated knockdown efficiency of transcription factors NRF2, SP1, ELK1, and CREB in HCT116 cells. Cells were transfected with individual siRNAs for 48 h. Data are presented as mean ± SD (*n* = 3). *p < 0.05 vs siNC. (b) GPX4 mRNA levels measured by qPCR following knockdown of each transcription factor and treatment with the HDAC3 inhibitor RGFP966 (10 μM, 24 h). Among the tested factors, only NRF2 knockdown significantly attenuated the RGFP966-induced changes in GPX4 expression, suggesting that NRF2 is required for HDAC3-mediated regulation of GPX4. Data are shown as mean ± SD (*n* = 3). *p < 0.05. (c, d) NRF2 mRNA (c) and protein (d) levels after HDAC3 knockdown (shHDAC3) or RGFP966 treatment (10 μM, 24 h) in HCT116 cells. Both interventions led to significant downregulation of NRF2. β-actin was used as the loading control. Data represent three independent experiments. *p < 0.05.

HCT116 cells were transfected with siRNAs targeting each factor, followed by treatment with the HDAC3-specific inhibitor RGFP966. Knockdown efficiency was confirmed by qPCR (Fig. 3a). Notably, only NRF2 knockdown significantly abolished the RGFP966-induced upregulation of GPX4 (Fig. 3b), whereas silencing of SP1, ELK1, or CREB had no such effect. These findings suggest that NRF2 is a critical downstream effector of HDAC3 in regulating GPX4 transcription.

To further determine whether HDAC3 modulates NRF2 at the transcriptional level, we measured NRF2 mRNA and protein levels after HDAC3 knockdown or RGFP966 treatment. Both interventions significantly reduced NRF2 expression (Figs. 3c–3d), indicating that HDAC3 positively regulates NRF2 transcription, which in turn maintains GPX4 expression.

Previous studies in vascular smooth muscle cells have shown that HDAC3 represses NRF2 transcription by binding to its promoter in cooperation with FOXM1, leading to histone H3 deacetylation and chromatin condensation [10]. Although this mechanism has not been directly validated in colorectal cancer (CRC), our findings suggest that HDAC3 may similarly regulate NRF2 expression at the epigenetic level in CRC cells, thereby modulating downstream targets such as GPX4 and contributing to ferroptosis resistance.

Together, these findings delineate an HDAC3–NRF2–GPX4 axis that governs ferroptosis resistance in colorectal cancer cells. This axis highlights a novel epigenetic mechanism through which HDAC3 modulates ferroptosis sensitivity, offering potential therapeutic targets for CRC treatment.

### GPX4 Is Functionally Required for Ferroptosis Sensitization Induced by HDAC3 Inhibition

**Fig. 4.**
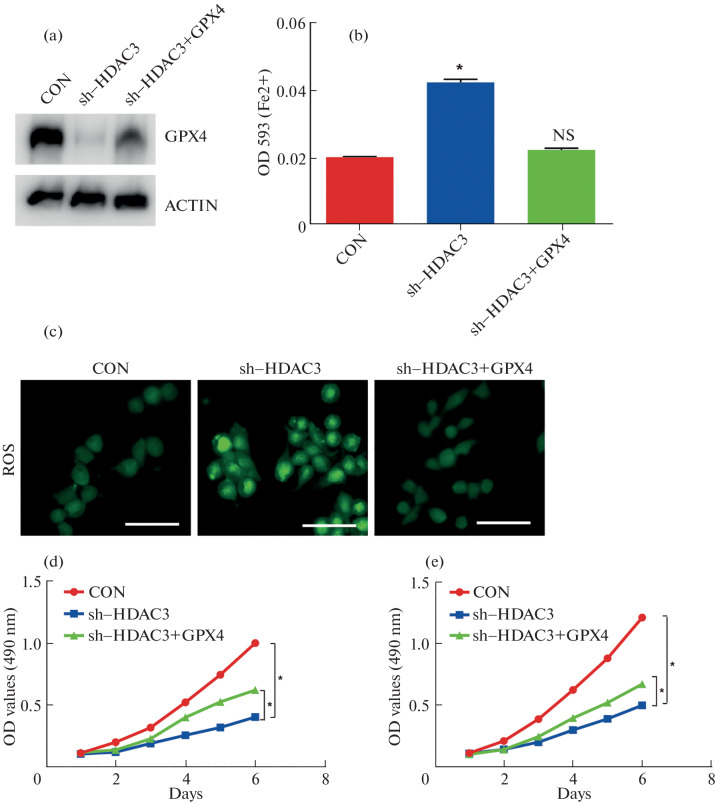
GPX4 is functionally required for ferroptosis sensitization induced by HDAC3 inhibition in colorectal cancer cells. (a) Western blot confirming GPX4 overexpression in HCT116 cells with HDAC3 knockdown. Cells were transduced with a FLAG-tagged GPX4 construct. ACTIN served as a loading control. Blots are representative of three independent experiments. (b) Intracellular ferrous iron (Fe^2+^) levels measured by colorimetric assay. HDAC3 knockdown increased Fe^2+^ accumulation, which was significantly reduced upon GPX4 re-expression. Data are shown as mean ± SD (*n* = 3). *p < 0.05. (c) Lipid ROS levels assessed using CellROX™ Green Reagent. GPX4 overexpression attenuated lipid ROS accumulation induced by HDAC3 knockdown. Representative fluorescence images are shown; quantification performed using ImageJ. (d) Cell viability evaluated by CCK-8 assay. GPX4 re-expression rescued the HDAC3 knockdown-induced suppression of cell growth. Data represent mean ± SD (*n* = 3). *p < 0.05. (e) Treatment with the ferroptosis inhibitor Ferrostatin-1 (Fer-1, 1 μM) phenocopied the effect of GPX4 overexpression. Co-treatment with Fer-1 in HDAC3-deficient cells significantly decreased Fe^2+^ accumulation and restored cell viability, indicating that HDAC3 loss promotes ferroptosis via a GPX4-dependent mechanism. *p < 0.05 vs shHDAC3 + vehicle.

To investigate whether GPX4 suppression is functionally involved in HDAC3-mediated ferroptosis sensitization, we performed rescue experiments by overexpressing GPX4 in HDAC3-silenced HCT116 cells. Western blot analysis confirmed successful re-expression of GPX4 (Fig. 4a).

Functionally, GPX4 overexpression significantly mitigated the ferroptosis-associated phenotypes induced by HDAC3 knockdown. Specifically, elevated intracellular Fe^2+^ levels were markedly reduced upon GPX4 restoration (Fig. 4b). In parallel, lipid ROS accumulation was attenuated, and cell viability was partially rescued, as assessed by CCK-8 assay (Figs. 4c–4d).

These findings indicate that GPX4 contributes to the ferroptosis sensitivity observed upon HDAC3 loss. To further confirm ferroptosis dependence, we treated cells with the ferroptosis inhibitor ferrostatin-1 (Fer-1). Fer-1 treatment phenocopied GPX4 overexpression, reducing iron accumulation and lipid peroxidation in HDAC3-deficient cells (Figure 4E), supporting the notion that HDAC3 loss primarily promotes ferroptosis through a GPX4-regulated pathway. Together, these results suggest that GPX4 acts as a key mediator of ferroptosis downstream of HDAC3, although additional regulatory components may also be involved. These data strengthen the link between HDAC3 activity and ferroptosis resistance in colorectal cancer cells. 

## DISCUSSION

In this study, we identify histone deacetylase 3 (HDAC3) as a key epigenetic suppressor of ferroptosis in colorectal cancer (CRC) and delineate a novel HDAC3–NRF2–GPX4 regulatory axis that governs ferroptotic sensitivity. Our findings reveal that HDAC3 transcriptionally sustains the expression of NRF2, a master antioxidant transcription factor, which in turn promotes GPX4 expression—an essential enzyme that protects cells from lipid peroxidation. Pharmacological inhibition or genetic silencing of HDAC3 leads to downregulation of NRF2 and GPX4, thereby triggering iron accumulation, oxidative lipid damage, and ferroptotic cell death.

### HDAC3 Serves as a Ferroptosis Gatekeeper in CRC

While HDAC3 has been widely studied in the context of chromatin remodeling, gene repression, and tumorigenesis, our findings extend its role to ferroptosis regulation in CRC. Among various ferroptosis-related genes, only GPX4 was consistently and significantly downregulated following HDAC3 inhibition. This identifies HDAC3 as a lineage-specific ferroptosis regulator in CRC, contrasting with its pro-ferroptotic role reported in other cancer types such as hepatocellular carcinoma or glioblastoma. These results underscore the context-dependent duality of HDAC3 function in cancer.

### HDAC3 Controls GPX4 Transcription via NRF2

Mechanistically, we show that HDAC3 regulates GPX4 expression by modulating NRF2 at the transcriptional level. Knockdown of NRF2 abrogated GPX4 expression even under HDAC3 inhibition, confirming its role as a key transcriptional intermediary. Although prior work in vascular disease contexts has shown that HDAC3 suppresses NRF2 transcription via promoter repression, our data in CRC cells suggest the opposite—namely, that HDAC3 supports NRF2 expression. This highlights a potential context-specific switch in HDAC3-mediated transcriptional control and suggests that its regulation of NRF2 is finely tuned by tissue-specific epigenetic cues.

### GPX4 Is a Functional Effector of HDAC3-Mediated Ferroptosis Resistance

The rescue experiments further validate GPX4 as a critical functional effector downstream of HDAC3. GPX4 overexpression reversed HDAC3 knockdown-induced ferroptotic phenotypes, including iron overload, ROS accumulation, and impaired proliferation. Moreover, treatment with ferrostatin-1 mimicked this rescue effect, confirming the ferroptotic nature of the observed cell death. These results consolidate GPX4 as a central node through which HDAC3 maintains ferroptosis resistance in CRC.

### Other HDACs Implicated in Ferroptosis

Beyond HDAC3, recent studies have begun to uncover roles for other class I and II HDACs in ferroptosis regulation. HDAC1 and HDAC2, often functioning redundantly in chromatin remodeling, have been reported to influence ferroptosis sensitivity. For example, HDAC1 has been shown to regulate ferroptosis sensitivity, primarily via lactylation at K412. In colorectal cancer models, HDAC inhibitors reduce this lactylation, promote FSP1 mRNA degradation, and enhance lipid peroxidation and ferroptotic death in HCT116 xenografts [11]. HDAC2 has also been implicated in ferroptosis regulation, although most mechanistic studies have been performed in non-colorectal models. In nasopharyngeal carcinoma, HDAC2 was shown to enhance ACSL4 acetylation, thereby promoting ferroptosis and radiosensitivity [12]. These findings suggest that HDAC2 may also play a pro-ferroptotic role, albeit in a cancer-type specific manner.

In contrast, HDAC6—a cytoplasmic class IIb deacetylase—has been shown to suppress ferroptosis, but in the context of acute liver failure rather than cancer. In histone H3‑stimulated ferroptosis of hepatic macrophages, HDAC6 activates the NOD2–NF‑κB pathway and thereby alleviates lipid peroxidation and ferroptotic cell death [13].

Taken together with our current findings on HDAC3, these studies highlight the isoform-specific and context-dependent roles of HDACs in ferroptosis regulation. Combinatorial targeting of select HDACs may represent a promising strategy to enhance ferroptosis sensitivity and overcome therapeutic resistance in cancer.

### Implications for Cancer Therapy

Our study provides a new rationale for targeting HDAC3 in CRC to overcome ferroptosis resistance. Combining HDAC3 inhibitors with ferroptosis inducers such as RSL3 or Erastin could represent a promising therapeutic strategy to eliminate treatment-refractory cancer cells. Given HDAC3’s additional roles in DNA repair, cell cycle progression, and immune regulation, dual targeting strategies may also synergize with immunotherapy or chemoradiation.

Importantly, the HDAC3–NRF2–GPX4 axis may also serve as a predictive biomarker of ferroptosis sensitivity in CRC, and potentially in other cancers. Future studies should explore whether HDAC3 expression levels correlate with resistance to ferroptosis-inducing agents in clinical samples.

### Limitations and Future Directions

While our study reveals a novel regulatory pathway, several questions remain. First, in vivo validation using xenograft or genetically engineered mouse models is needed to confirm the physiological relevance of the HDAC3–NRF2–GPX4 axis. Second, we cannot exclude the involvement of other transcriptional regulators or epigenetic modifiers in this pathway. Third, metabolic rewiring and tumor microenvironmental cues such as hypoxia or immune infiltration may modulate ferroptosis sensitivity downstream of HDAC3.

In conclusion, our findings define a novel HDAC3–NRF2–GPX4 signaling axis that epigenetically governs ferroptosis in CRC. This discovery not only enhances our mechanistic understanding of ferroptosis regulation but also unveils HDAC3 as a promising therapeutic target for ferroptosis-based intervention in colorectal cancer.

## CONCLUSIONS

In summary, our study identifies HDAC3 as a pivotal epigenetic regulator that suppresses ferroptosis in colorectal cancer (CRC). Mechanistically, HDAC3 maintains redox balance by sustaining NRF2 transcription, which in turn promotes GPX4 expression—an essential antioxidant enzyme that protects against ferroptotic stress. This newly defined HDAC3–NRF2–GPX4 axis underpins the ability of CRC cells to resist ferroptotic cell death.

Rescue experiments confirm that the ferroptosis-sensitizing effects of HDAC3 inhibition are largely mediated through GPX4 downregulation, underscoring GPX4 as a key functional effector downstream of HDAC3. These findings expand the known roles of HDAC3 in cancer biology and reveal ferroptosis regulation as a previously unrecognized dimension of its oncogenic activity.

From a therapeutic perspective, targeting HDAC3— particularly in combination with ferroptosis inducers such as RSL3 or Erastin – could offer a selective strategy to eradicate treatment-resistant CRC cells. Given HDAC3’s broader functions in chromatin remodeling and tumor progression, this approach may also be applicable to other HDAC3-driven malignancies.

Future studies should aim to validate this regulatory pathway in vivo and assess the clinical potential of HDAC3-based combination therapies. Additionally, dissecting the interplay between HDAC3, immune signaling, and iron metabolism may further refine ferroptosis-targeted strategies and accelerate their translation into oncology practice.

## Footnotes

1 # The corresponding author.

## References

[1] Xi, Y. and Xu, P., Global colorectal cancer burden in 2020 and projections to 2040, *Transl. Oncol.*, 2021, vol. 14, p. 101174. 10.1016/j.tranon.2021.101174 PMC8273208 34243011

[2] Gao, W., Wang, X., Zhou, Y., Wang, X., and Yu, Y., Autophagy, ferroptosis, pyroptosis, and necroptosis in tumor immunotherapy, *Signal Transduct. Target Ther.*, 2022, vol. 7, p. 196. 35725836 10.1038/s41392-022-01046-3 PMC9208265

[3] Cheng, X., Zhao, F., Ke, B., Chen, D., and Liu, F., Harnessing ferroptosis to overcome drug resistance in colorectal cancer: promising therapeutic approaches, *Cancers* (Basel), 2023, vol. 15. 10.3390/cancers15215209 PMC10649072 37958383

[4] Zhang, C., Liu, X., Jin, S., Chen, Y., and Guo, R., Ferroptosis in cancer therapy: a novel approach to reversing drug resistance, *Mol. Cancer*, 2022, vol. 21, p. 47. 35151318 10.1186/s12943-022-01530-y PMC8840702

[5] Yao, X. et al., Emerging roles of energy metabolism in ferroptosis regulation of tumor cells, *Adv. Sci.* (Weinh.), 2021, vol. 8, p. e2100997. 10.1002/advs.202100997 PMC8596140 34632727

[6] Forcina, G.C. and Dixon, S.J., GPX4 at the crossroads of lipid homeostasis and ferroptosis, *Proteomics*, 2019, vol. 19, p. e1800311. 10.1002/pmic.201800311 30888116

[7] Song, X., Wang, X., Liu, Z., and Yu, Z., Role of GPX4-mediated ferroptosis in the sensitivity of triple negative breast cancer cells to gefitinib, *Front. Oncol.*, 2020, vol. 10, p. 597434. 10.3389/fonc.2020.597434 PMC7785974 33425751

[8] Yang, M., Luo, H., Yi, X., Wei, X., and Jiang, D.S., The epigenetic regulatory mechanisms of ferroptosis and its implications for biological processes and diseases, *MedComm*, 2023, vol. 4, no. 3, p. e267. 10.1002/mco2.267 PMC10203370 37229485

[9] Zhan, W. et al., USP38 regulates the stemness and chemoresistance of human colorectal cancer via regulation of HDAC3, *Oncogenesis*, 2020, vol. 9, p. 48. 32404892 10.1038/s41389-020-0234-z PMC7220910

[10] Chen, F. et al., Inhibiting HDAC3 (histone deacetylase 3) aberration and the resultant Nrf2 (nuclear factor erythroid-derived 2-related factor-2) repression mitigates pulmonary fibrosis, *Hypertension*, 2021, vol. 78, pp. e15–e25. 34148362 10.1161/HYPERTENSIONAHA.121.17471

[11] Yang, Z., Lactylation of HDAC1 confers resistance to ferroptosis in colorectal cancer, *Adv. Sci.* (Weinh.), 2025, vol. 12, p. e2408845. 10.1002/advs.202408845 PMC11947995 39888307

[12] Zhou, P., HAT1/HDAC2 mediated ACSL4 acetylation confers radiosensitivity by inducing ferroptosis in nasopharyngeal carcinoma, *Cell Death Dis.*, 2025, vol. 16, p. 160. 40050614 10.1038/s41419-025-07477-4 PMC11885570

[13] Chen, Q., NOD2-mediated HDAC6/NF-kappab signalling pathway regulates ferroptosis induced by extracellular histone H3 in acute liver failure, *J. Cell Mol. Med.*, 2022, vol. 26, pp. 5528–5538. 36226351 10.1111/jcmm.17582 PMC9639038

